# Literary Notes

**Published:** 1909-09-25

**Authors:** 


### LITERARY NOTES.

Messrs. A. and C. Black are reissuing a cheaper edition of "The Letters of Dr. John Brown, with Letters from Ruskin, Thackeray, and Others," edited by his son and D. W. Forrest, D.D., and containing a biographical introduction by Elizabeth T. McLaren.

The new subject index of the London Library will prove worth the attention of some medical men. Compiled by Dr. Hagberg Wright (brother of Sir Almroth Wright), and secretary to this institution, it is a valuable piece of work quite worthy the premier British library of general reference. To those who subscribe before October 1 the price is 25s.

